# Corrigendum: Hepatic p63 regulates steatosis via IKKβ/ER stress

**DOI:** 10.1038/ncomms16059

**Published:** 2017-06-16

**Authors:** Begoña Porteiro, Marcos F. Fondevila, Teresa C. Delgado, Cristina Iglesias, Monica Imbernon, Paula Iruzubieta, Javier Crespo, Amaia Zabala-Letona, Johan Fernø, Bárbara González-Terán, Nuria Matesanz, Lourdes Hernández-Cosido, Miguel Marcos, Sulay Tovar, Anxo Vidal, Julia Sánchez-Ceinos, Maria M. Malagon, Celia Pombo, Juan Zalvide, Arkaitz Carracedo, Xabier Buque, Carlos Dieguez, Guadalupe Sabio, Miguel López, Patricia Aspichueta, María L. Martínez-Chantar, Ruben Nogueiras

Nature Communications 8: Article number:15111; DOI: 10.1038/ncomms15111 (2017); Published: 05 08 2017; Updated: 06 16 2017

The affiliation details for Paula Iruzubieta and Javier Crespo are incorrect in this Article. The correct affiliation details for these authors are given below:

Department of Gastroenterology and Hepatology, Marqués de Valdecilla University Hospital, Centro de Investigación Biomédica en Red de Enfermedades Hepáticas y Digestivas (CIBERehd). Infection, Immunity and Digestive Pathology Group, Research Institute Marqués de Valdecilla (IDIVAL), Santander, Spain.

