# Supplementary material for: Hepatic p63 regulates steatosis via IKKβ/ER stress
Source: Nat Commun. 2017 May 8;8:15111. doi: 10.1038/ncomms15111 (PMC5424198; doi:10.1038/ncomms15111)
Supplement: Supplementary Information — Supplementary figures, supplementary tables, and supplementary references. [file ncomms15111-s1.pdf]

Supplementary table 1. Primers and probes used for gene amplification.

NAME	5'3' PRIMER
**18S**	FW CGG CTA CCA CAT CCA AGG AA<br>RV GCT GGA ATT ACC GCG GCT<br>PB GAC GGC AAG TCT GGT GCC AGC A
**hHPRT**	FW TTATGGACAGGACTGAACGTCTTG<br>RV CCAGCAGGTCAGCAAAGAATT
**mHPRT**	Commercial primers provided by Applied biosystems (Ref. Mm01545399_m1)
**Arginase**	FW TCC ACC CTG ACC TAT GTG TCA TT<br>RV CCT GGT ACA TCT GGG AAC TTT CC<br>PB ACA TCA ACA CTC CCC TGA CAA CCA GCT CT
**F480**	Commercial primers provided by Applied biosystems (Ref. Mm00802529m1)
**HsIKKβ**<br><br><br>**MmIKKβ**	FW GAC ATT GCC TCT GCG CTT AGA TA<br>RV CTT GCT GCA GGA CGA TGT TTT<br>PB AAC AGA ATC ATC CAT CGG GAT CTA AAG CCA<br><br>FW TGCAGGACACTGTGAAGGAG<br>RV CTGGCAGAGTGAGATGTCCA
**IL6**	Commercial primers provided by Applied biosystems (Ref.Mm00446190m1)
**LPL**	FW GGGAAATGATGTGGCCAGATT<br>RV CCCTAAGAGGTGGACGTTGTCT<br>PB ACTGGATGGAGGAGGAGTTTAACTACCCCC
**MAC2**	FW CCA ACG CAA ACA GGA TTGTTCT<br>RV CCT GCT TCG TGT TAC ACA CAA TG<br>PB ATG TTG CCT TCC ACT TTA ACC CCC GC
**MTTP1**	FW CACCTGGCCACCACTGTTCT<br>RV GGTGGTATATCCTGTTCAAGGTCTTC<br>PB ATGTCTCCTTCATCACAGATGAGGTG
**NOS2**	FW TGA CGC TCG GAA CTG TAG CA<br>RV TGA AGT CAT GTT TGC CGT CAC T<br>PB CAA TGG CAA CAT CAG GTC GGC CA
**p53**	Commercial primers provided by Applied biosystems (Ref.Mm01731287_m1)
**p63**	Commercial primers provided by Applied biosystems (Ref.Hs00978343_m1)
**PGC1α**	FW CGATCACCATATTCCAGGTCAAG<br>RV CGATGTGTGCGGTGTCTGTAGT<br>PB AGGTCCCCAGGCAGTAGATCCTCTTCAAGA
**PPARγ**	FW CGTGCTGCGGTACAGCC<br>RV CGCCAACAGCTTCTCCTTCTC<br>PB ATGTCTCACAATGCCATCAGGTTTGGGC
**SCARB1**	FW CCG ACC CTG TGT TGT CAG AAG<br>RV ATC CCA GTG ACC GGA TGG AT<br>PB TGG TCT GAA CCC TAA CCC AAA GGA GCA T
**TNFα**	Commercial primers provided by Applied biosystems (Ref. Mm99999068m1)
**XBP1S**	Commercial primers provided by Applied biosystems (Ref. Mm99999068m1)
**CPT1**	FW ATC ATG TAT CGC CGC AAA CT<br>RV ATC TGG TAG GAG CAC ATG GGC
**ACADM**	FW AGGTTTCAAGATCGCAATGG<br>RV CTCCTTGGTGCTCCACTAGC
**ACADL**	FW GCATCAACATCGCAGAGAAA<br>RV GGCTATGGCACCGATACACT
**FATP2**	FW ATGCCGTGTCCGTCTTTTAC<br>RV GACCTGTGGTTCCCGAAGTA

Supplementary table 2. Antibodies used for western blot.

Peptide/protein target	Name of Antibody	Manufacter, catalog #	Species raisedin; monoclonal or polyclonal	Dilution used
**Anti-Apolipoprotein B**	**ApoB**	Abcam, Cambridge, UK; ab-20737	Rabbit polyclonal	1:500
**Fatty acid synthase**	FAS (H-300)	Santa Cruz Biotechnology, Santa Cruz, CA, USA; sc-20140	Rabbit polyclonal	1:1000
**BAX**	BAX	Cell Signaling Technology, Hitchin, Herts, UK; #2772	Rabbit polyclonal	1:1000
**c-Jun N terminal kinases 1/3**	JNK 1/3 (C-17)	Santa Cruz Biotechnology, Santa Cruz, CA, USA; sc-474	Rabbit polyclonal	1:1000
**phospho-SAPK/JNK (Thr183/Tyr185) (81E11)**	phospho-SAPK/JNK (Thr183/Tyr185) (81E11)	Cell Signaling Technology, Hitchin, Herts, UK; #4668	Rabbit monoclonal	1:1000
**Anti-IRE 1**	IRE 1	Abcam, Cambridge, UK; ab-37073	Rabbit polyclonal	1:1000
**IRE 1 alpha phosphoespecific [ser 724]**	phospho-IRE 1-alpha [ser 724]	Novus Biologicals, Littleton CO, USA. NB100-2323	Rabbit polyclonal	1:1000
**X-box binding protein-1**	XBP1	Santa Cruz Biotechnology, Santa Cruz, CA, USA; sc-7160	Rabbit polyclonal	1:1000
**Phospho-PERK (Thr 981)**	phospho-PERK	Santa Cruz Biotechnology, Santa Cruz, CA, USA; sc-32577	Rabbit polyclonal	1:1000
**Eucariotic initiation complex subunit alpha**	eiF2α (FL-315)	Santa Cruz Biotechnology, Santa Cruz, CA, USA; sc-11386	Rabbit polyclonal	1:1000
**phospho-eiF2α**	phospho-eiF2α (Ser 52)	Santa Cruz Biotechnology, Santa Cruz, CA, USA; sc-101670	Rabbit polyclonal	1:1000
**Caspase-3**	Caspase-3 (8G10)	Cell Signaling Technology, Hitchin, Herts, UK; #9665	Rabbit monoclonal	1:1000
**Cleaved caspase 3**	Cleaved caspase-3 (Asp 175) (5A1E)	Cell Signaling Technology, Hitchin, Herts, UK; #9664	Rabbit monoclonal	1:1000
**Caspase 7**	Caspase 7	Cell Signaling Technology, Hitchin, Herts, UK; #9492	Rabbit polyclonal	1:1000
**Cleaved caspase 7**	Cleaved caspase 7 (Asp 198)	Cell Signaling Technology, Hitchin, Herts, UK; #9491	Rabbit polyclonal	1:1000
**TAp63α**	p63 (TA) clone 6189	Biolegend CA,USA; cat: 618902	Rabbit polyclona.	1:1000
**ΔNp63α**	p63 (ΔN) clone 6190)	Biolegend, CA, USA; cat: 619002	Rabbit polyclona.	1:1000
**p63 (H-129)**	p63	Santa Cruz Biotechnology, Santa Cruz, CA, USA; sc-11386	Rabbit polyclonal.	1:1000
**Anti-p73 [EP436Y]**	P73	Abcam, Cambridge, UK; ab-40658)	Rabbit monoclonal	1:1000
**Anti-SHC (phosphor S36) [6E10]**	p66shc	Abcam, Cambridge, UK; ab-54518	Mouse monoclonal	1:1000
**p21 (C19)**	p21	Santa Cruz, CA, USA; sc-397	Rabbit polyclonal	1:1000
**IκB kinases α and β**	Phosphor-IKKα/β (Ser180/Ser181)-R	Santa Cruz, CA, USA; sc-23470-R	Rabbit polyclonal	1:1000
**Anti IKK β (phosphor Y188)**	Phospho IKK β	Abcam, Cambridge, UK; ab-194519	Rabbit polyclonal	1:1000
**Anti-IKK β [EPR6043]**	IKK β	Abcam, Cambridge, UK; ab-124957	Rabbit monoclonal	1:1000
**Glyceraldehyde-3-phosphate dehydrogenase (GAPDH)**	GAPDH (6C5)	Merck Millipore, Darmstadt, Germany; CB1001	Mouse monoclonal	1:5000
**Transferrin (I-20)**	Transferrin	Santa Cruz, CA, USA; sc-22597		
**Polyclonal Rabbit Anti-Mouse Immunoglobulins/HRP**	Anti-mouse	DAKO (Agillent Technologies Company), Denmark. P0260		1:5000
**Polyclonal Goat Anti-Rabbit Immunoglobulins/HRP**	Anti-rabbit	DAKO (Agillent Technologies Company), Denmark. P0448		1:5000
**GFP**	GFP	Living Colors 632381	Rabbit monoclonal	1:10000

Supplementary Table 3. Characteristics of patients and controls used for p63 mRNA expression.

Variable	Obese patients with NAFLD (n = 35)	Controls (n =11)	*P*
**Age (years)**	43.7 (11.4)	50.4 (17.5)	0.140
**Female:male ratio**	29:9	7:4	0.451
**Hypertension (n)**	15 (39.5)	2 (18.2)	0.287
**Diabetes mellitus (n)**	8 (21.1)	0 (0)	0.172
**BMI (kg/m^2)**	49.2 (6.9)	27.2 (4.24)	< 0.001*
**Fasting blood sugar (mg/dL)**	104.2 (34.4)	93.67 (14.3)	0.376
**AST (IU/L)**	24.8 (13.4)	21.0 (3.8)	0.137
**ALT (IU/L)**	30.8 (17.6)	28.4 (12.6)	0.703
**Bilirubin (mg/dL)**	0.43 (0.2)	0.49 (0.27)	0.383
**Total cholesterol (mg/dL)**	199.1 (33.6)	207.0 (37.3)	0.563
**Triglycerides (mg/dL)**	129.9 (50.0)	97.6 (38.3)	0.096
**LDL-cholesterol (mg/dL)**	118.7 (35.6)	124.8 (36.0)	0.668
**HDL-cholesterol (mg/dL)**	49.0 (13.2)	62.7 (18.5)	0.020*
**NAS score**	4.9 (1.5)	0	< 0.001*
**Steatosis**	2.1 (0.9)	0	< 0.001*
**Lobular inflammation**	1.6 (0.8)	0	< 0.001*
**Hepatocyte ballooning**	1.4 (0.6)	0	< 0.001*

Variables are presented as mean (standard deviation) or absolute frequency (percentage) and are compared by means of Student's T test or χ^2 test. NAFLD: non alcoholic fatty liver disease. BMI: body mass index. AST: aspartate aminotransferase. ALT: alanine aminotransferase. NAS: NAFLD Activity Score. NAFLD (NAS score <4; n = 8) and NASH (NAS score >4; n = 27).

Supplementary Table 4. Characteristics of patients and controls used for TAp63α immunohistochemistry.

NUMBER OF PACIENTS	N= 39
GENDER (FEMALE/MALE)	21/18
MEAN AGE (YEARS)	45 ± 11
NAS SCORE 1-3 (N)	22
AVERAGE	2.4 ± 1.0
NAS SCORE ≥ 4	17
AVERAGE	4.5 ± 1.0

Surgically resected specimens of well-characterized patients were used. Histological scoring was performed according to the NASH Clinical Research Network criteria (NASH CRN)[18].

Supplementary Table 5. Logistic regression between TAp63α and parameters contributing to the NAS score.

	Chi Square	*P*	Odds Ratio (95% CI)
**NAS**	5.7651	***0.0163***	1.5367 (1.0527; 2.2433)
**Steatosis**	4.8200	***0.0281***	1.9128 (1.0473; 3.4935)
**Inflammation**	2.3527	0.1251	2.1669 (0.7719; 6.0831)
**Balooning**	1.3474	0.2457	1.5406 (0.7430; 3.1943)
**Fibrosis**	0.9782	0.3226	1.5602 (0.6424; 3.7893)

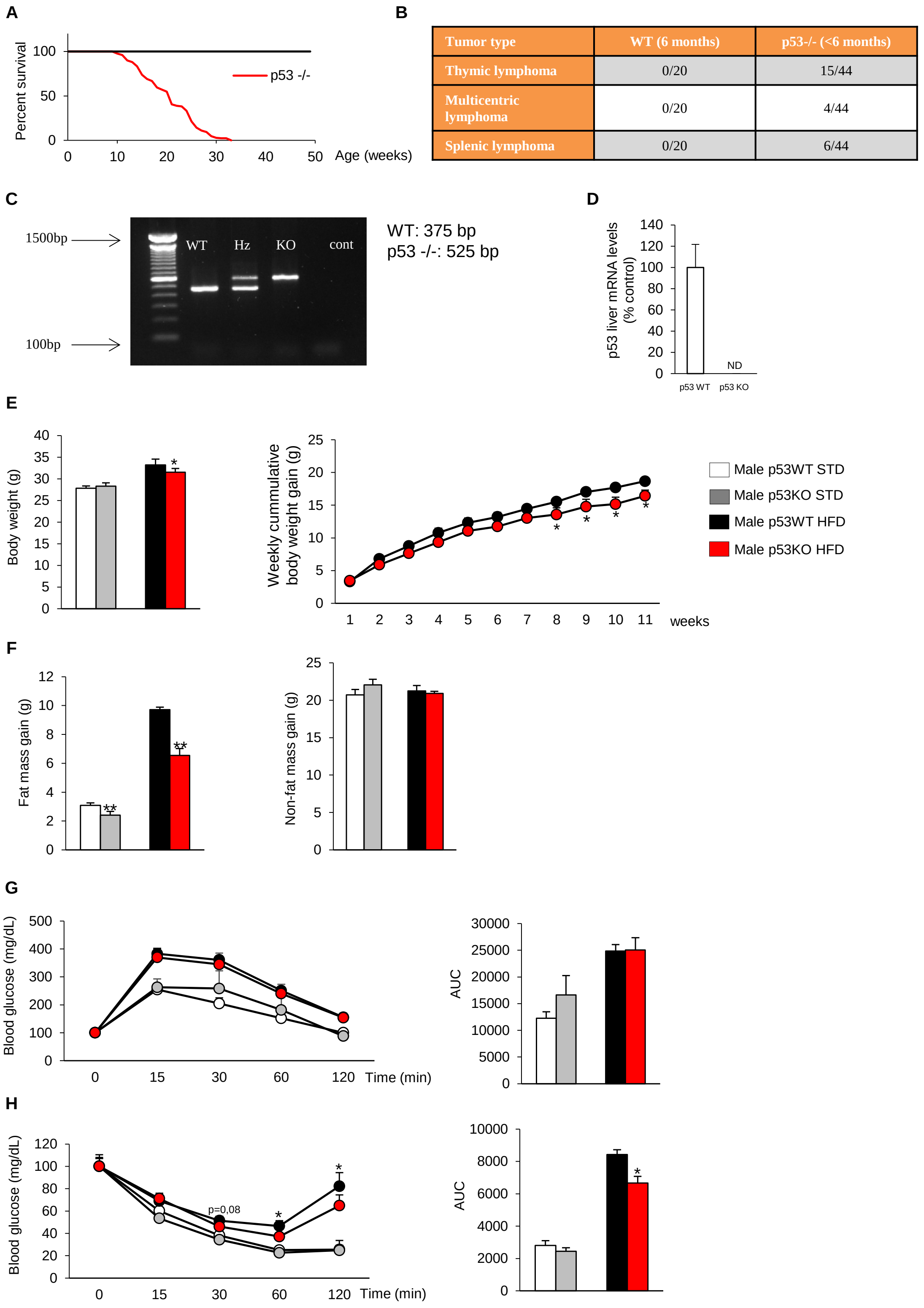

Tumor type	WT (6 months)	p53-/- (<6 months)
Thymic lymphoma	0/20	15/44
Multicentric lymphoma	0/20	4/44
Splenic lymphoma	0/20	6/44

Supplementary figure 1. Metabolic phenotype of male p53 deficient mice fed with chow or high fat diet. (A) Representative lifespan (% survival) of WT and p53 null mice. (B) Tumour spectrum of WT and p53 null mice. (C) Products of PCR for p53

gene in WT and p53 null mice. (D) Liver p53 mRNA levels in WT and p53 null mice. HRPT were used to normalize mRNA levels. ND: non detected. (E) Body weight of male mice after free access to chow diet or high fat diet during 11 weeks. (F) Fat mass and non-fat mass gain. (G) Glucose tolerance test in male p53 null mice fed a chow diet or high fat diet. (H) Insulin tolerance test in male p53 null mice fed a chow diet or high fat diet. Data are presented as mean ± standard error mean (s.e.m.). Statistical differences are denoted by *p<0.05 and **p<0.01 (n = 9 WT and n = 5 KO in chow diet; n = 8 WT and n = 11 KO in high fat diet) using Student t-test comparing mice fed a chow diet or HDF as separated experiments.

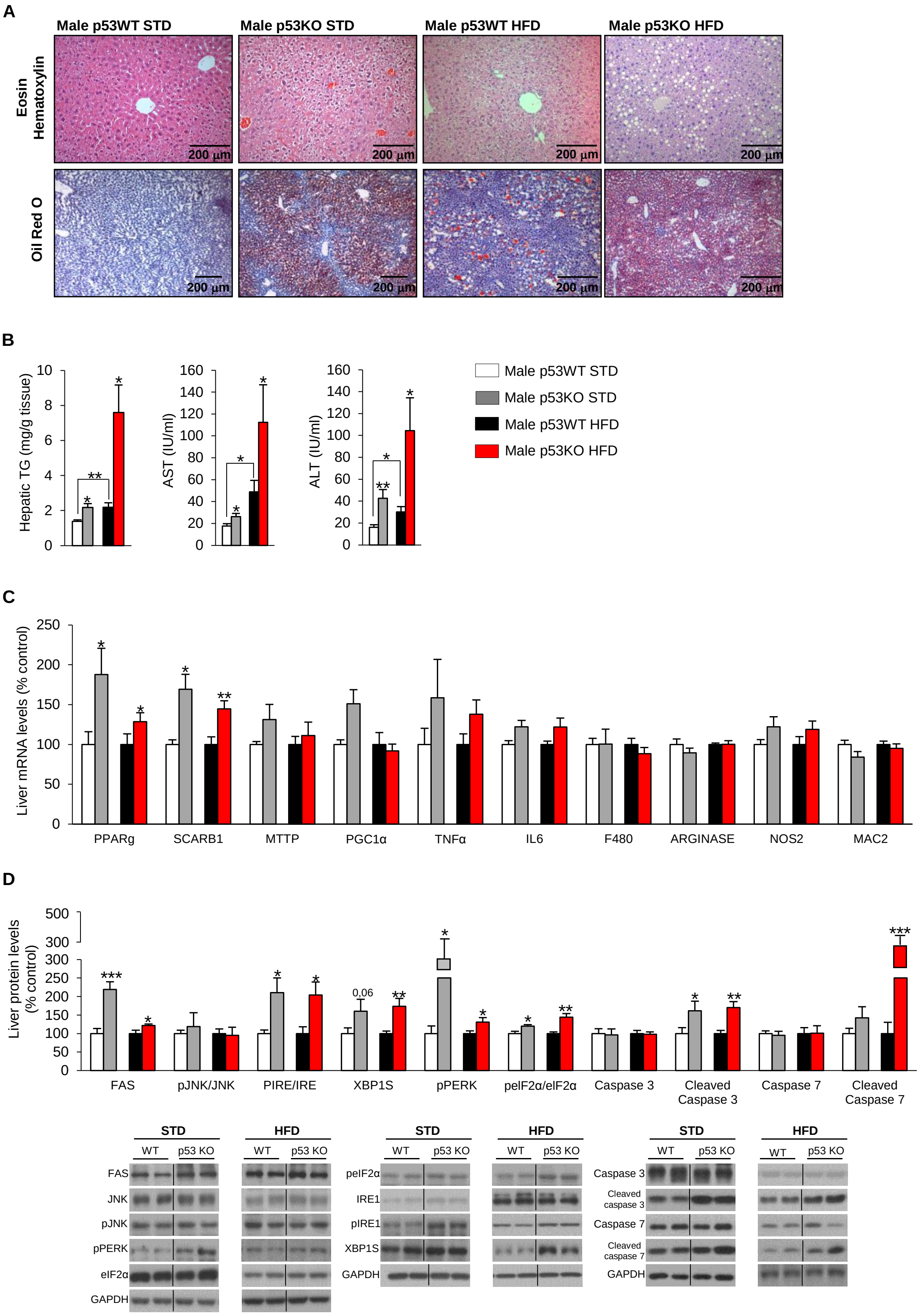

Supplementary figure 2. Effect of p53 deficiency on liver steatosis in mice fed chow or high fat diet (HFD). (A) Representative photomicrographs of haematoxylin-eosin (upper panel) and oil red O staining (lower panel) of mice liver sections (n = 4 per

group).(B) Total liver triglyceride content (TG) (n = 7 WT and KO chow diet mice; n = 7 WT and 9 KO HFD mice), and serum AST and ALT levels (n = 7 WT and 8 KO chow diet mice; n = 6 WT and 11 KO HFD mice); (C) liver mRNA levels of PPARγ, SCARB1, MTTP, PGC1α, TNFα, IL6, F480, arginase, NOS2 and MAC2, (n = 7 WT and 7 KO chow diet mice; n = 9 WT and 6 KO HFD mice). HRPT were used to normalize mRNA levels; (D) Liver protein levels of FAS, pJNK/JNK, pIRE/IRE, XBP1s, pPERK, pEIF2α/eIF2α, caspase 3, cleaved caspase 3, caspase 7 and cleaved caspase 7 in WT and global p53 KO mice fed with chow or HFD during 11 weeks. Protein GAPDH levels were used to normalize protein levels (n = 7 per group). Western blots were performed separately in mice fed a STD and mice fed a HFD, and the values of WT mice were always normalized to 100%. Dividing lines indicate splicings in the same gel. Data are presented as mean ± standard error mean (s.e.m.). Statistical significance ,*$p<0.05$, **$p<0.01$. For multiple comparison (B) a one way ANOVA followed by Bonferroni or Kruskal-Wallis test was performed. Student t-test was used in the other panels.

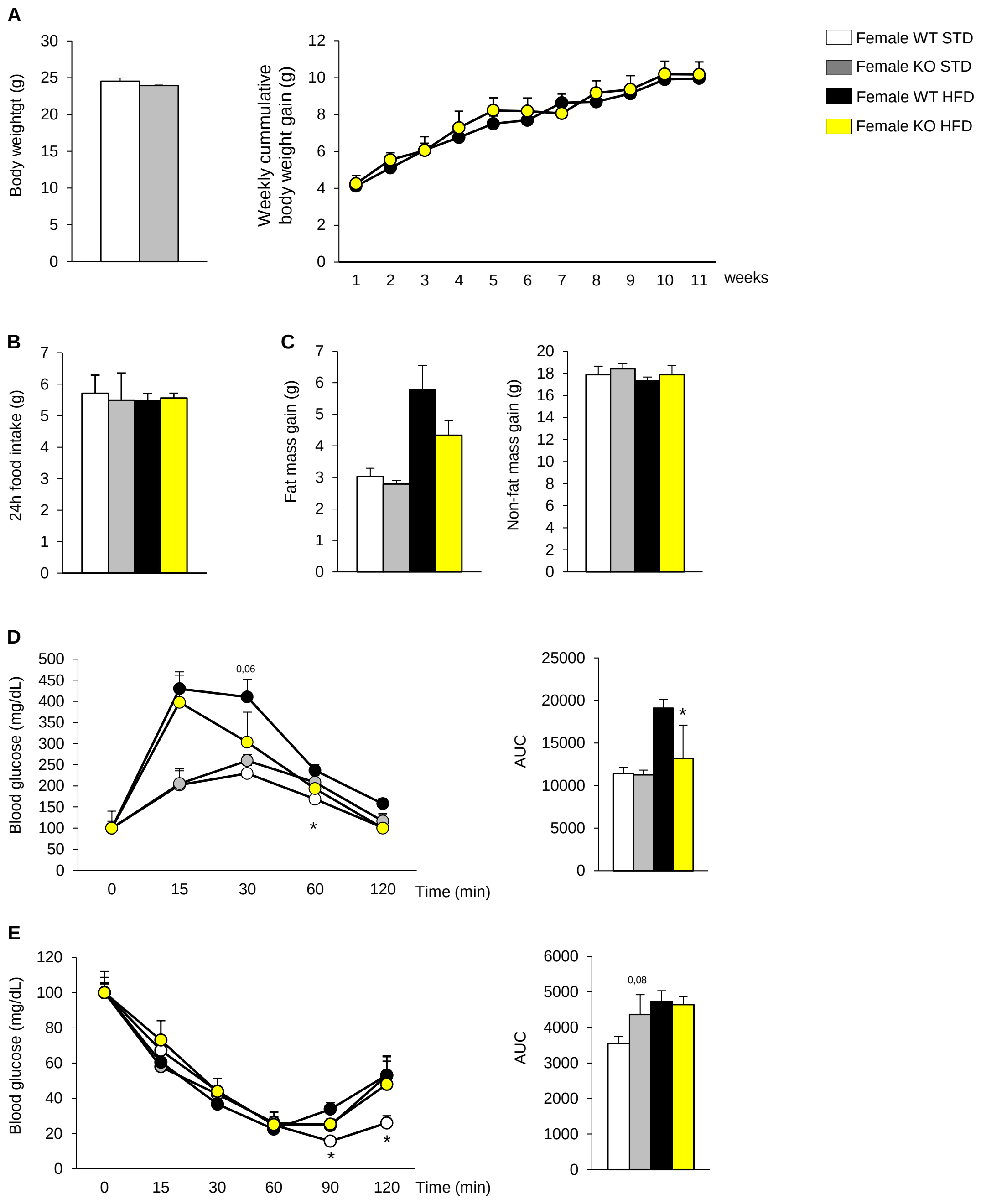

Supplementary figure 3. Metabolic phenotype of female p53 deficient mice fed a chow diet or high fat diet. (A) Body weight of female mice after free access to chow diet or high fat diet. (B) Cumulative food intake over 24h. (C) Fat mass and non-fat mass. (D) Glucose tolerance test in female p53 null mice fed a chow diet or high fat diet. (E) Insulin tolerance test in female p53 null mice fed a chow diet or high fat diet. Data are presented as mean ± standard error mean (s.e.m.). Statistical differences are

denoted by *p<0.05 for a two-tailed Student t-test (n = 8 WT and n = 7 KO in chow diet; n = 9 WT and n = 9 KO in high fat diet) comparing mice fed with chow diet or HDF as separated experiments. For multiple comparison (B, C, D, E) a one way ANOVA followed by Bonferroni or Kruskal-Wallis test was performed. Student t-test was used in the other panels.

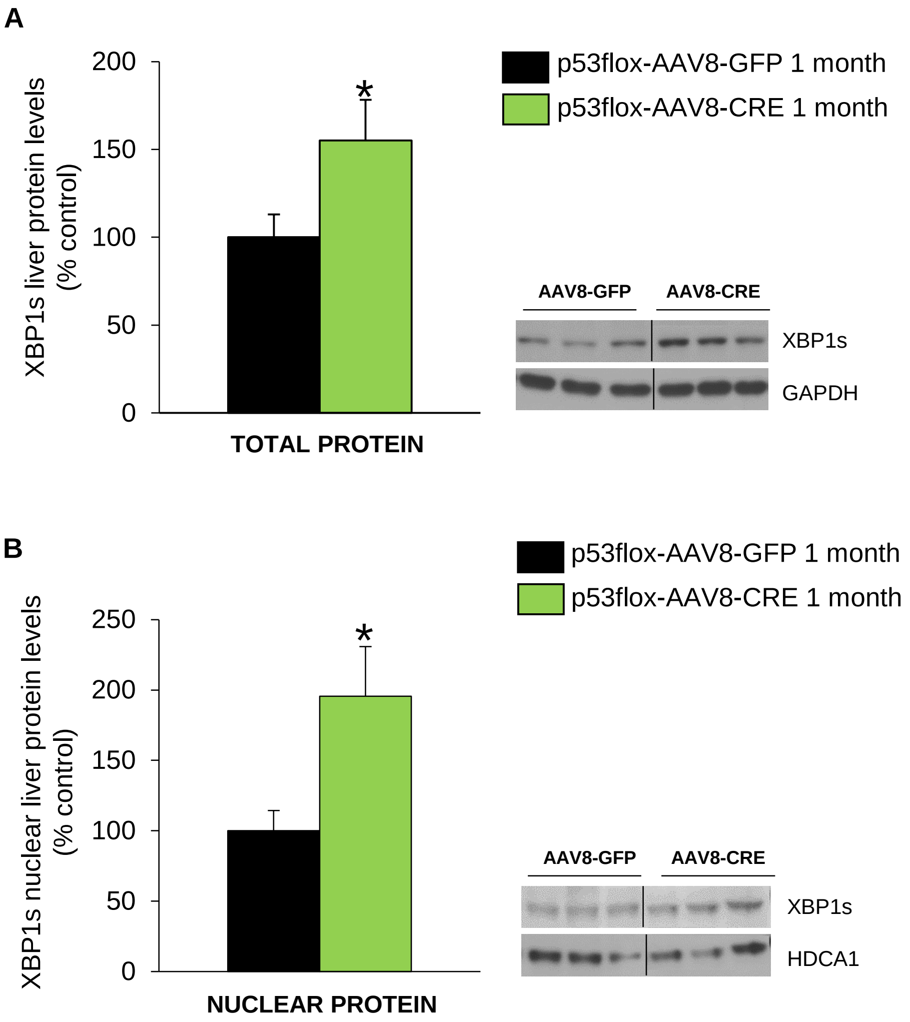

Supplementary figure 4. XBP1s is upregulated in the liver after hepatic p53 knockdown. (A) XBP1s western blot performed in total liver protein lysate. GAPDH was used to normalize protein levels (n = 7 per group). (B) XBP1s protein levels in liver following nuclear extraction. HDCA1 was used as loading control (n = 7 per group). Dividing lines indicate splicings in the same gel. Data are presented as mean ± standard error mean (s.e.m.). Statistical significance *p<0.05 and **p<0.01, was tested using Student t-test.

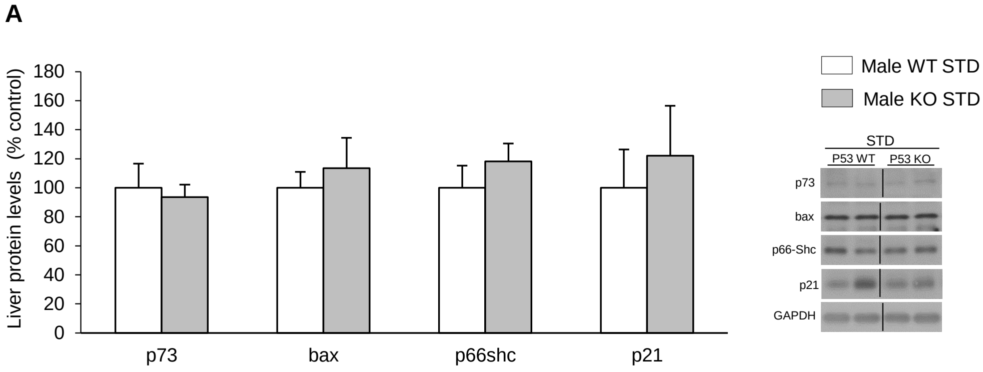

Supplementary figure 5. Expression of p53 downstream genes after the hepatic manipulation of p53. p73, bax, p66shc and p21 protein levels in WT and p53 null mice. GAPDH levels were used to normalize protein levels. Dividing lines indicate splicings in the same gel. Data are presented as mean ± standard error mean (s.e.m.). Statistical significance, *p<0.05 and **p<0.01, was tested using Student t-test.

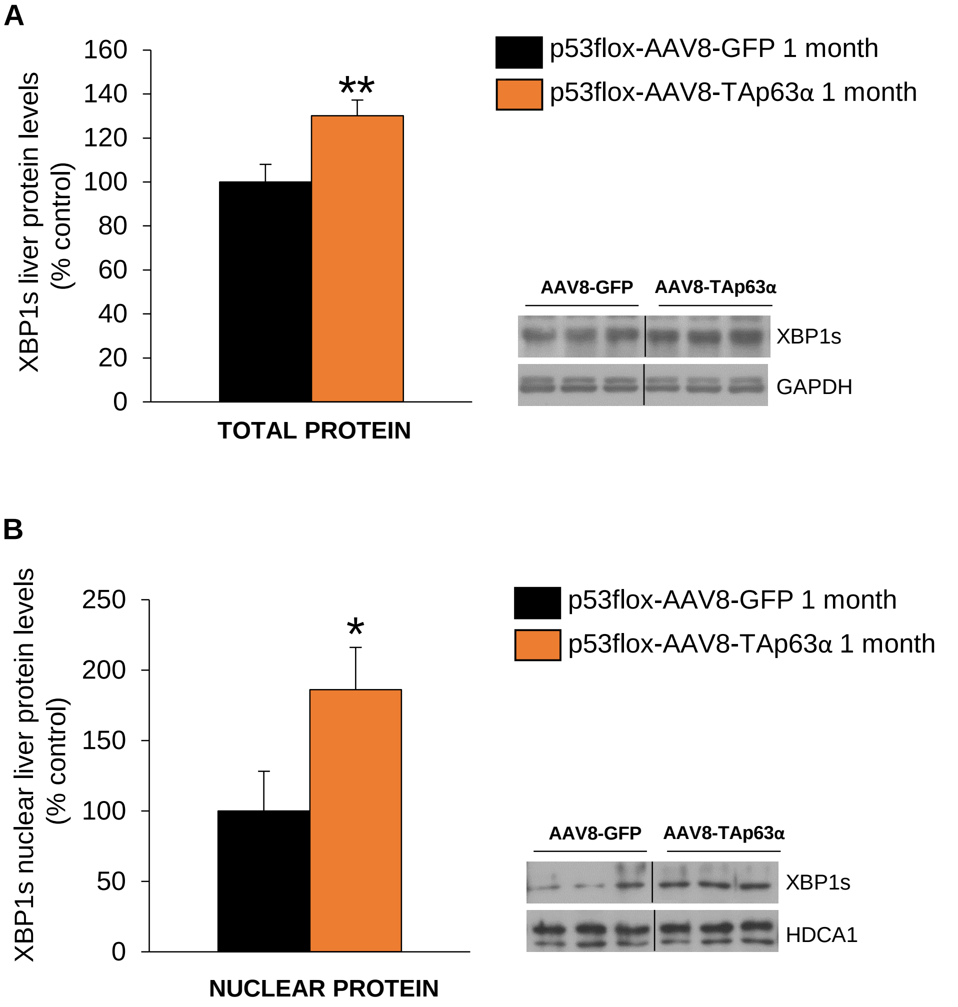

Supplementary figure 6. XPB1s expression increases after AAV8-Tap63α injection in mice. (A) XBP1s western blot in total liver protein lysate. GAPDH was used to normalize protein levels (n = 7 per group). (B) XBP1s protein levels in liver following nuclear extraction. HDCA1 was used as loading control (n = 7 per group). Dividing lines indicate splicings in the same gel. Data are presented as mean ± standard error mean (s.e.m.). Statistical significance *p<0.05 and **p<0.01, was tested using Student t-test.

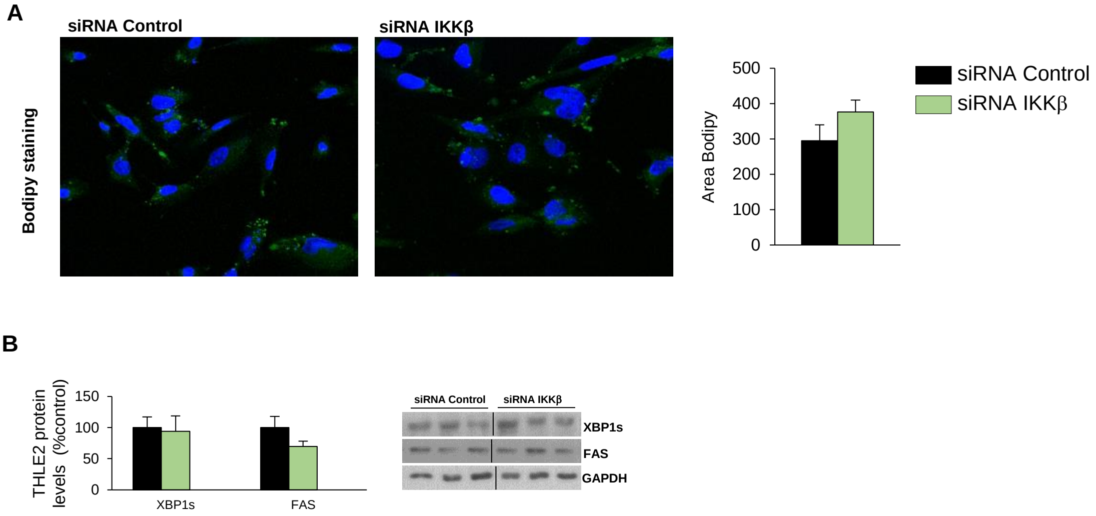

Supplementary figure 7. Effects of silencing IKKβ in THLE2 hepatocytes. (A) Representative dual channel fluorescent photomicrograph of THLE2 cells showing staining of lipids (green area, BODIPY 493/503) 24 hours after transfection with siRNA control (left image) and siRNA IKKβ (right image). Magnifications 63X. (B) Protein levels XBP1s and FAS in THLE2 cells after treatment with siRNA against either control or siRNA IKKβ (n = 3 per group). GAPDH was used to normalize protein levels. Data are presented as mean ± standard error mean (s.e.m.). Statistical differences are denoted by *p<0.05, **p<0.01 and ***p<0.001, was tested using Student t-test.

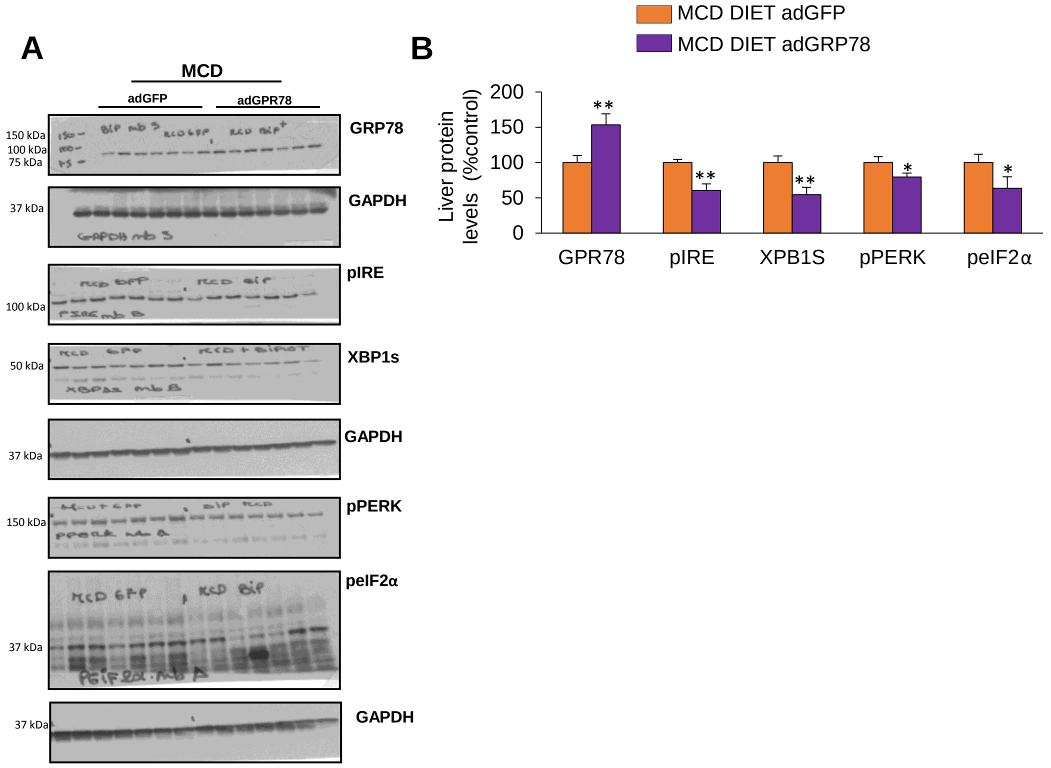

Supplementary figure 8. Hepatic GRP78 over-expression ameliorates methionine and choline deficient diet (MCD)-induced ER-stress. (A) Uncropped blots for protein levels of GPR78, pIRE, XBP1s, pPERK, peIF2α and GAPDH. (A) Protein levels after in the liver of mice fed a MCD-diet after the hepatic over-expression of GRP78. GAPDH levels were used to normalize protein levels (n = 7 per group). Data are presented as mean ± standard error mean (s.e.m.). *P < 0.05; **P < 0.01, was tested using Student t-test.

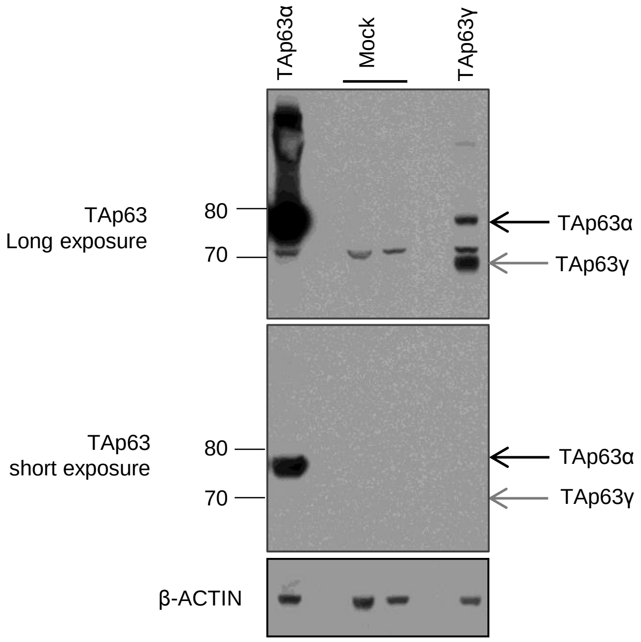

Supplementary figure 9. Over-expression of TAp63α in THLE2 cells and expression of TAp63α in human liver. Protein levels of TAp63γ in THLE2 cells after the over-expression of TAp63 γ. Data are presented as mean ± standard error mean (s.e.m.).

Uncropped blots for Figure 1A

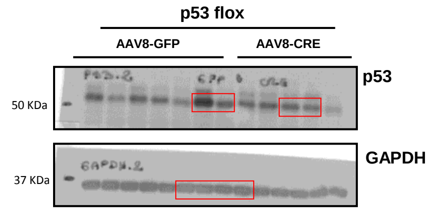

Uncropped blots for Figure 1D

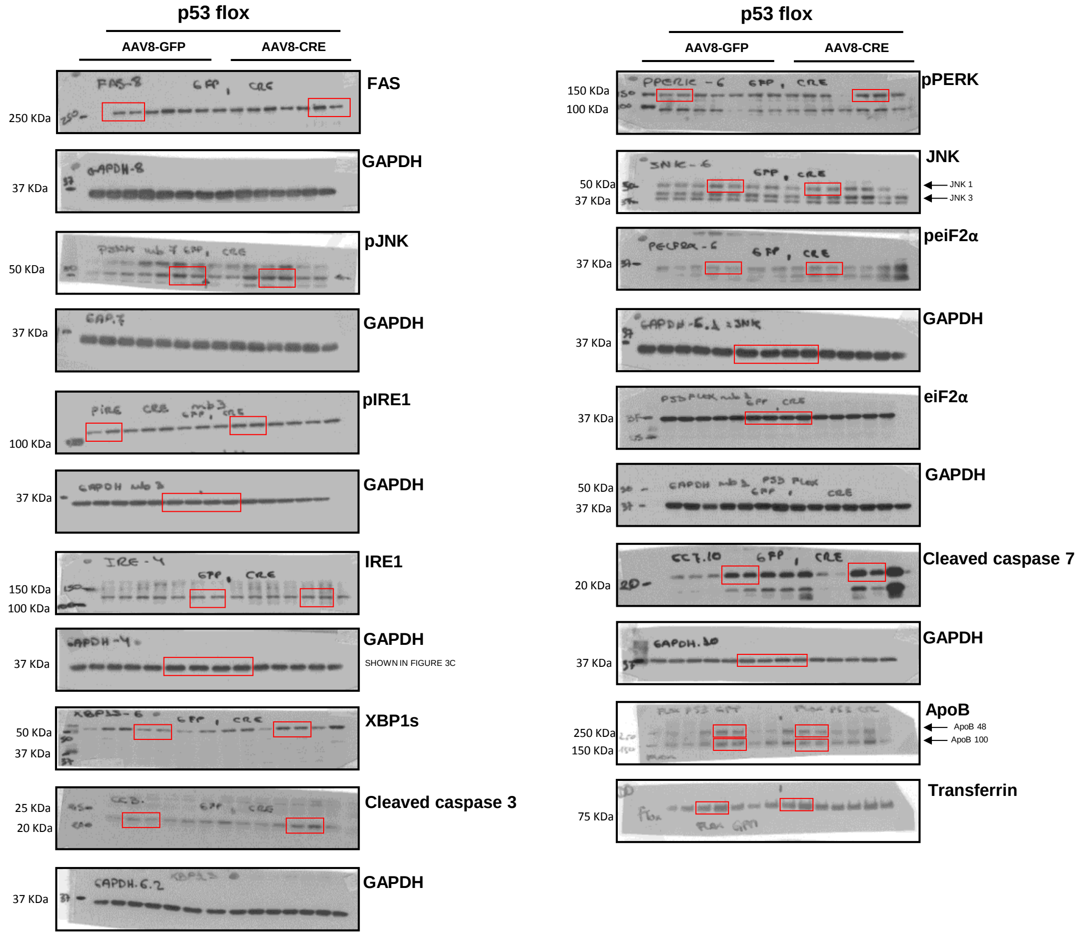

Please note that in Figure 1 not all GAPDH´s blots are shown in order to simplify the figure to the readers. Herein, we show the GAPDH for each blot and the bands used in the figure are marked in red squares.

Supplementary figure 10. Uncropped blots for figure 1A-1D.

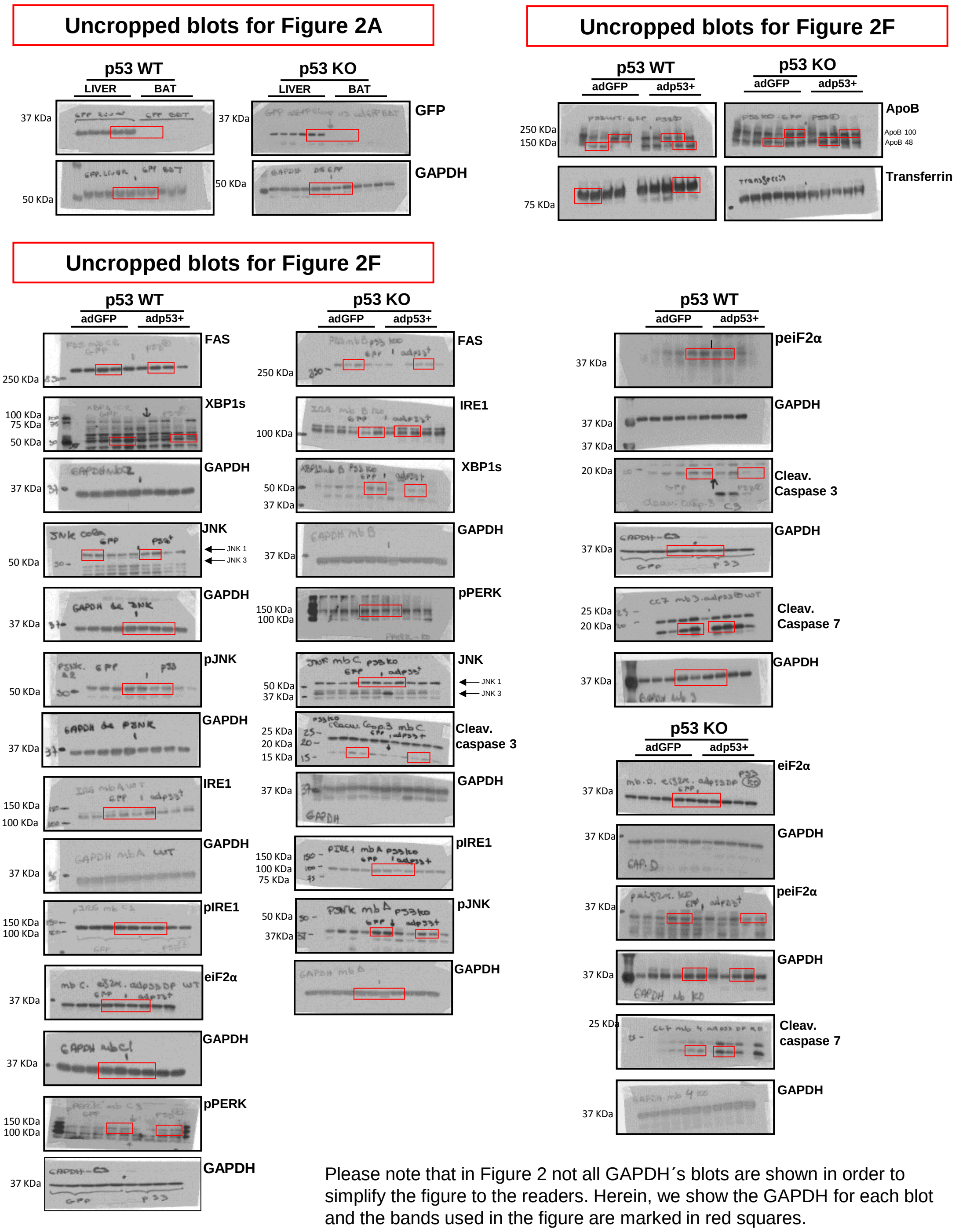

Please note that in Figure 2 not all GAPDH´s blots are shown in order to simplify the figure to the readers. Herein, we show the GAPDH for each blot and the bands used in the figure are marked in red squares.

Supplementary figure 11. Uncropped blots for figure 2A-2F.

Uncropped blots for Figure 3 A-B-C

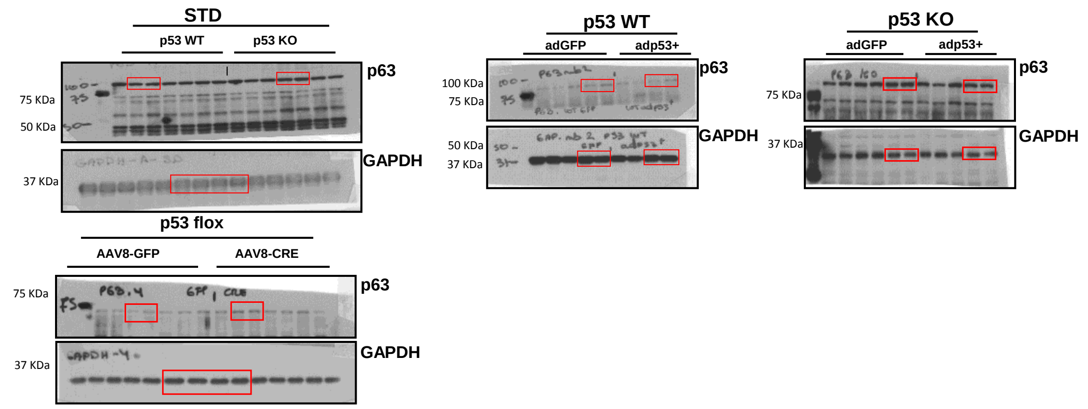

Uncropped blots for Figure 3 D

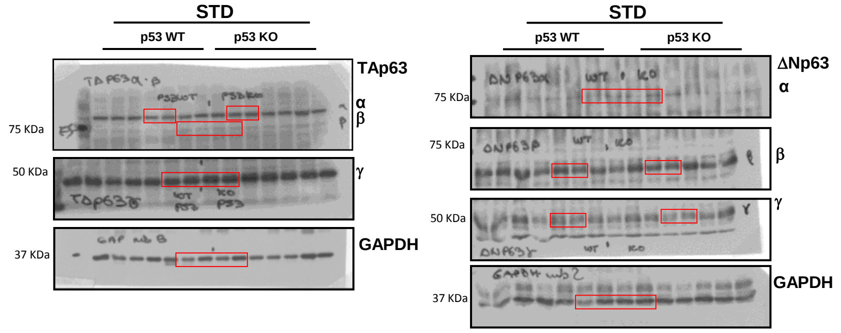

Uncropped blots for Figure 3 E

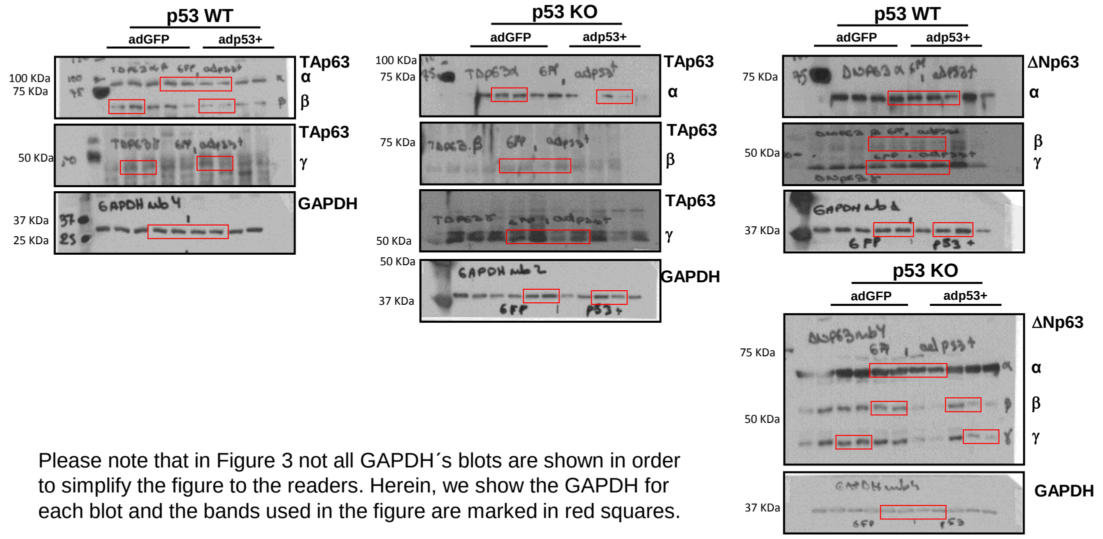

Please note that in Figure 3 not all GAPDH´s blots are shown in order to simplify the figure to the readers. Herein, we show the GAPDH for each blot and the bands used in the figure are marked in red squares.

Supplementary figure 12. Uncropped blots for figure 3.

Uncropped blots for Figure 4C

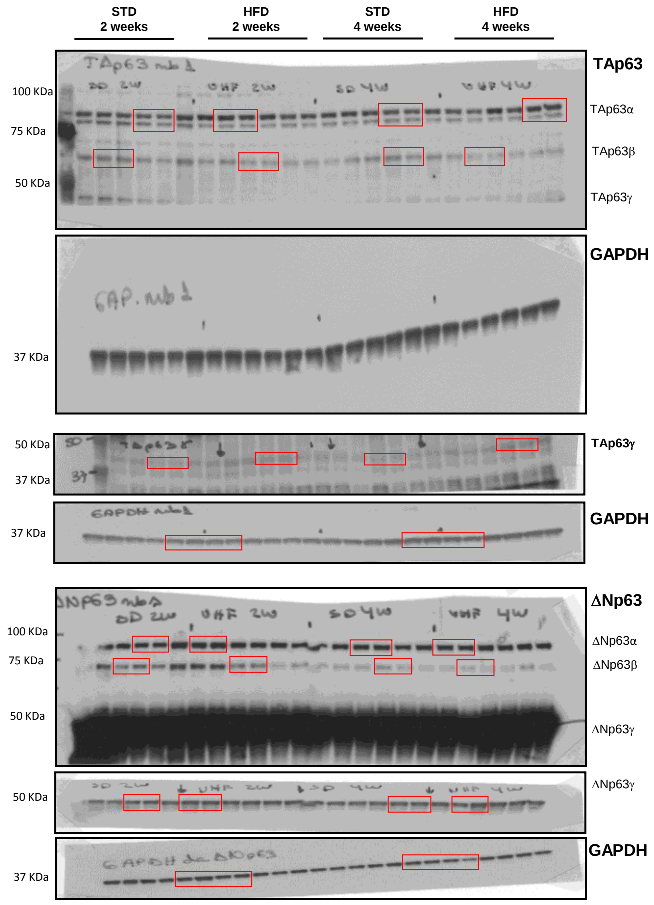

Uncropped blots for Figure 4D

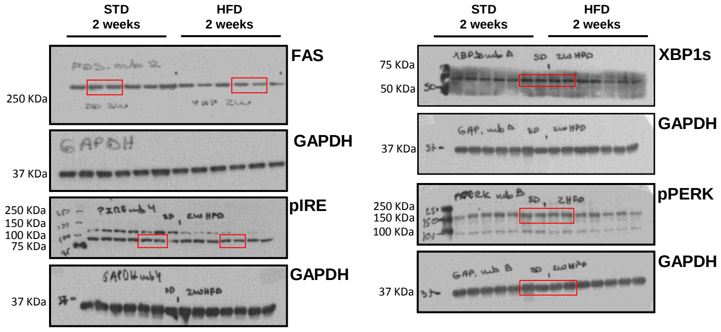

Please note that in Figure 4 not all GAPDH´s blots are shown in order to simplify the figure to the readers. Herein, we show the GAPDH for each blot and the bands used in the figure are marked in red squares.

Supplementary figure 13. Uncropped blots for figure 4C-4D.

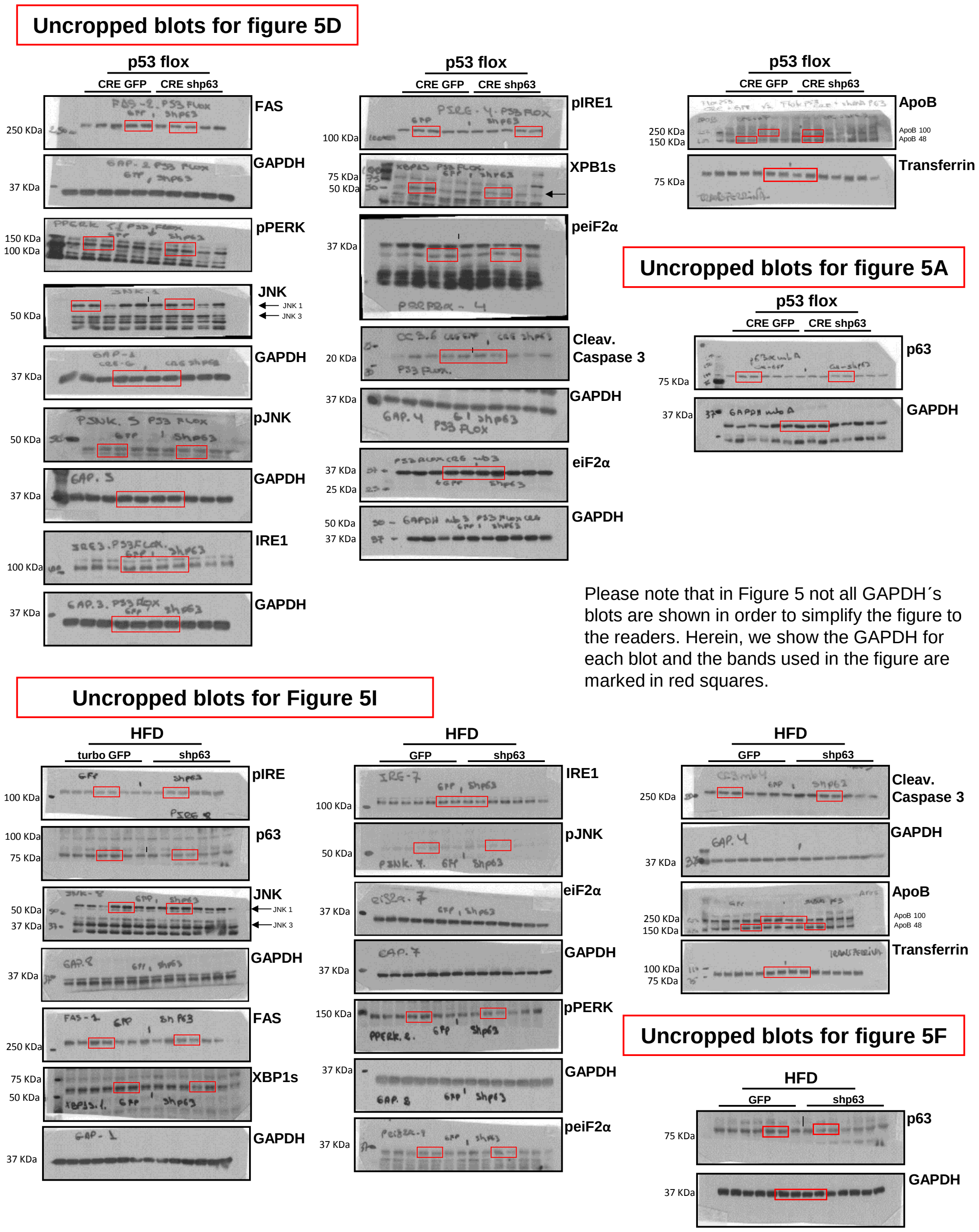

Please note that in Figure 5 not all GAPDH´s blots are shown in order to simplify the figure to the readers. Herein, we show the GAPDH for each blot and the bands used in the figure are marked in red squares.

Supplementary figure 14. Uncropped blots for figure 5A-5D-5F-5I.

Uncropped blots for Figure 6 A-D

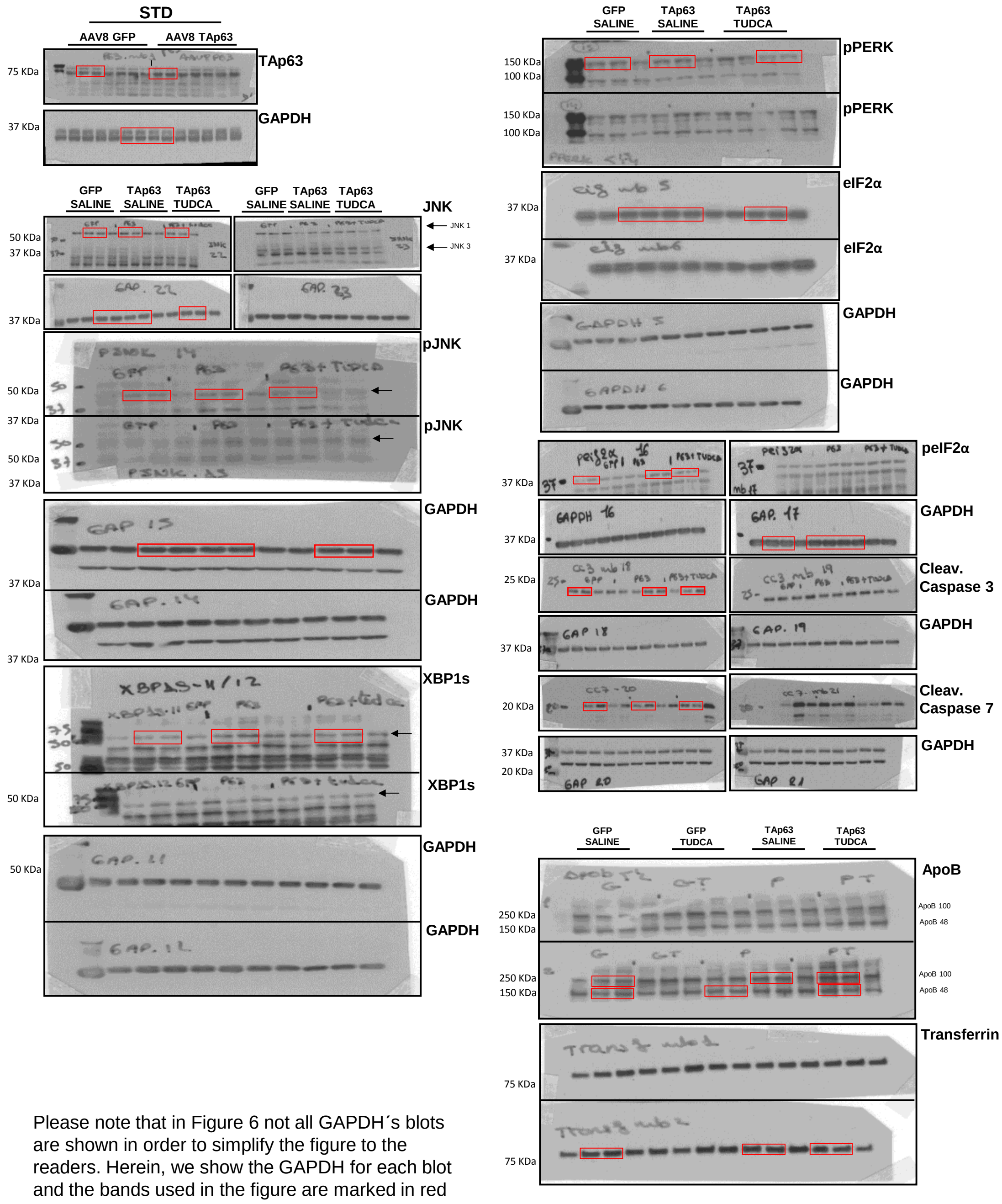

Please note that in Figure 6 not all GAPDH´s blots are shown in order to simplify the figure to the readers. Herein, we show the GAPDH for each blot and the bands used in the figure are marked in red squares.

Supplementary figure 15. Uncropped blots for figure 6A-D.

Uncropped blots for Figure 6 F

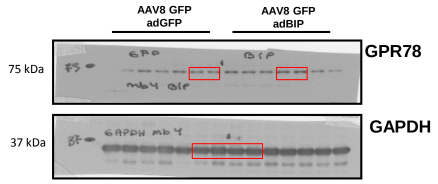

Uncropped blots for Figure 6 I

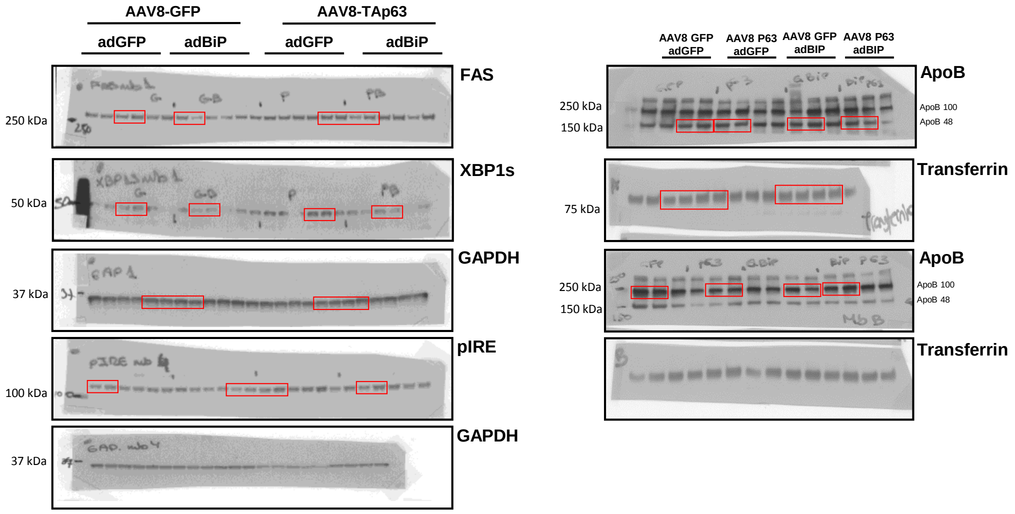

Please note that in Figure 6 not all GAPDH´s blots are shown in order to simplify the figure to the readers. Herein, we show the GAPDH for each blot and the bands used in the figure are marked in red squares.

Supplementary figure 16. Uncropped blots for figure 6F-I.

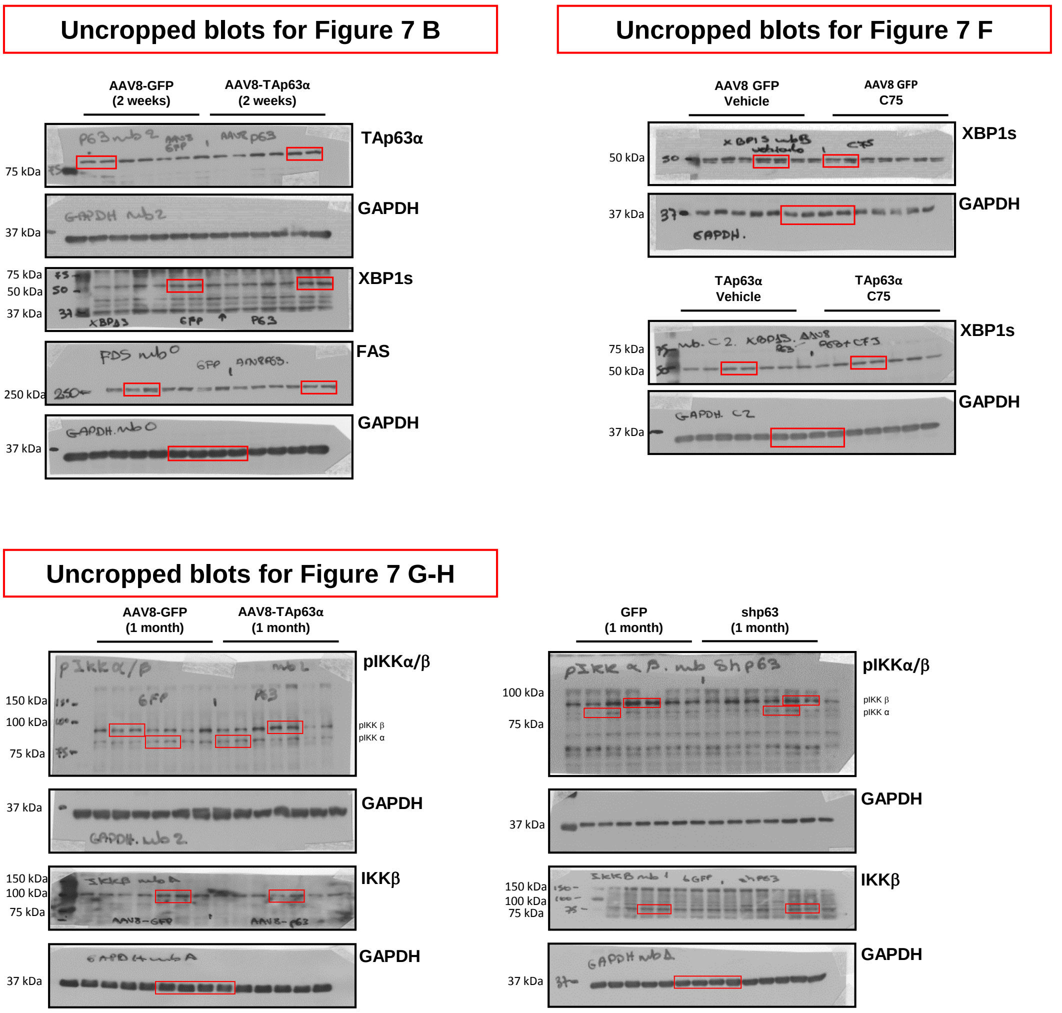

Please note that in Figure 7 not all GAPDH´s blots are shown in order to simplify the figure to the readers. Herein, we show the GAPDH for each blot and the bands used in the figure are marked in red squares.

Supplementary figure 17. Uncropped blots for figure 7B-7F-6G-7H.

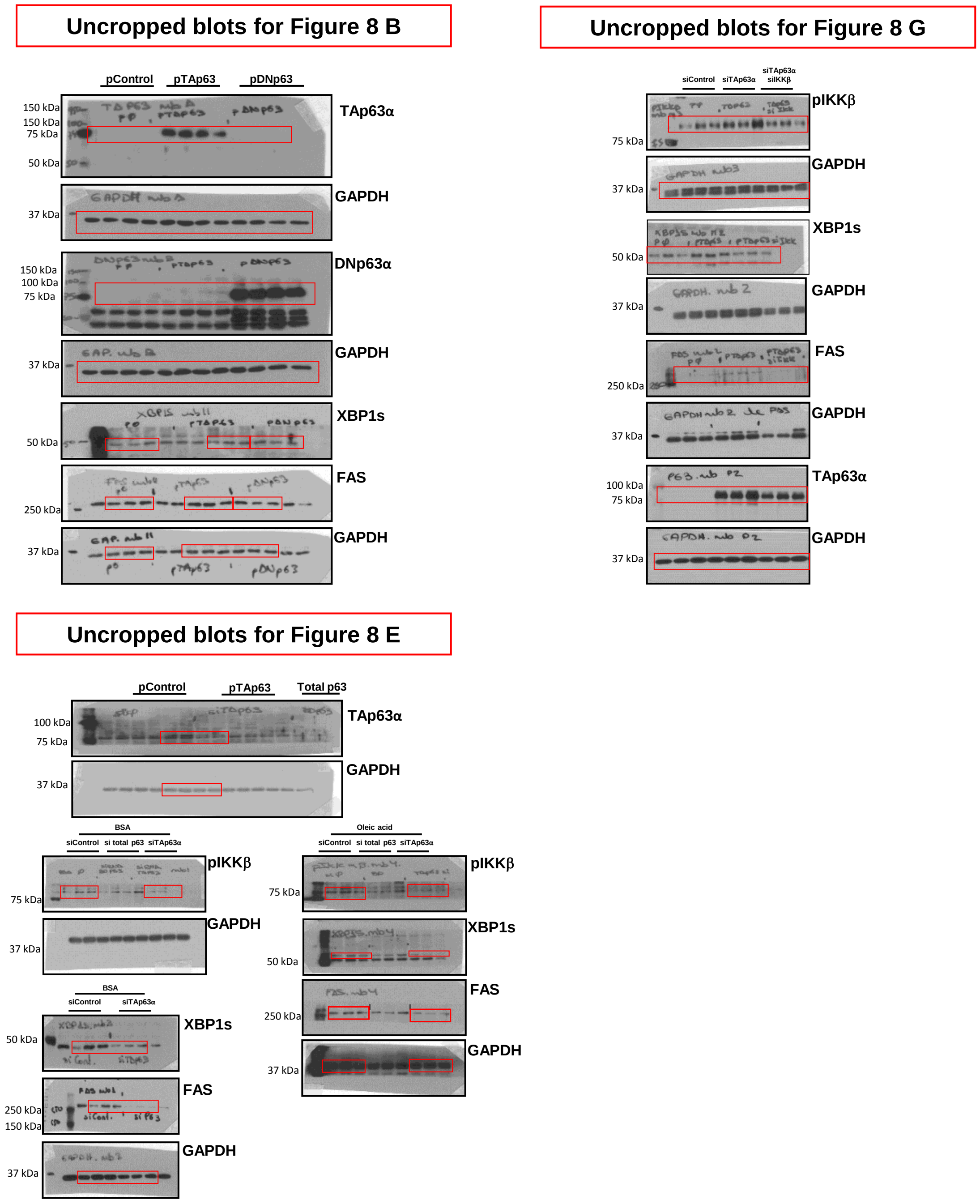

Please note that in Figure 8 not all GAPDH´s blots are shown in order to simplify the figure to the readers. Herein, we show the GAPDH for each blot and the bands used in the figure are marked in red squares.

Supplementary figure 18. Uncropped blots for figure 8B-8E-8G.

Supplementary References

1. Jacks, T., *et al.* Tumor spectrum analysis in p53-mutant mice. *Current biology : CB* **4**, 1-7 (1994).
2. Marino, S., Vooijs, M., van Der Gulden, H., Jonkers, J. & Berns, A. Induction of medulloblastomas in p53-null mutant mice by somatic inactivation of Rb in the

external granular layer cells of the cerebellum. *Genes & development* **14**, 994-1004 (2000).
3. Czyzyk, T.A., *et al.* kappa-Opioid receptors control the metabolic response to a high-energy diet in mice. *FASEB J* **24**, 1151-1159.
4. Nogueiras, R., *et al.* Direct control of peripheral lipid deposition by CNS GLP-1 receptor signaling is mediated by the sympathetic nervous system and blunted in diet-induced obesity. *J Neurosci* **29**, 5916-5925 (2009).
5. Imbernon, M., *et al.* Central melanin-concentrating hormone influences liver and adipose metabolism via specific hypothalamic nuclei and efferent autonomic/JNK1 pathways. *Gastroenterology* **144**, 636-649 e636 (2013).
6. Perez-Sieira, S., *et al.* Female Nur77-deficient mice show increased susceptibility to diet-induced obesity. *PloS one* **8**, e53836 (2013).
7. Gonzalez, C.R., *et al.* Regulation of visceral adipose tissue-derived serine protease inhibitor by nutritional status, metformin, gender and pituitary factors in rat white adipose tissue. *The Journal of physiology* **587**, 3741-3750 (2009).
8. Vazquez, M.J., *et al.* Central resistin regulates hypothalamic and peripheral lipid metabolism in a nutritional-dependent fashion. *Endocrinology* **149**, 4534-4543 (2008).
9. Velasquez, D.A., *et al.* The central Sirtuin 1/p53 pathway is essential for the orexigenic action of ghrelin. *Diabetes* **60**, 1177-1185.
10. Nakai, H., *et al.* Unrestricted hepatocyte transduction with adeno-associated virus serotype 8 vectors in mice. *Journal of virology* **79**, 214-224 (2005).
11. Wang, H.Q., *et al.* Positive feedback regulation between AKT activation and fatty acid synthase expression in ovarian carcinoma cells. *Oncogene* **24**, 3574-3582 (2005).
12. Aspichueta, P., Perez, S., Ochoa, B. & Fresnedo, O. Endotoxin promotes preferential periportal upregulation of VLDL secretion in the rat liver. *Journal of lipid research* **46**, 1017-1026 (2005).
13. Bligh, E.G. & Dyer, W.J. A rapid method of total lipid extraction and purification. *Canadian journal of biochemistry and physiology* **37**, 911-917 (1959).
14. Ruiz, J.I. & Ochoa, B. Quantification in the subnanomolar range of phospholipids and neutral lipids by monodimensional thin-layer chromatography and image analysis. *Journal of lipid research* **38**, 1482-1489 (1997).
15. Martinez-Una, M., *et al.* S-Adenosylmethionine increases circulating very-low density lipoprotein clearance in non-alcoholic fatty liver disease. *Journal of hepatology* **62**, 673-681 (2015).
16. Hirschey, M.D., *et al.* SIRT3 regulates mitochondrial fatty-acid oxidation by reversible enzyme deacetylation. *Nature* **464**, 121-125 (2010).
17. Vila-Brau, A., De Sousa-Coelho, A.L., Mayordomo, C., Haro, D. & Marrero, P.F. Human HMGCS2 regulates mitochondrial fatty acid oxidation and FGF21 expression in HepG2 cell line. *The Journal of biological chemistry* **286**, 20423-20430 (2011).
18. David E. Kleiner, E.M.B., 2 Mark Van Natta,3 Cynthia Behling,4 Melissa J. Contos,5 Oscar W. Cummings,6 Linda D. Ferrell,7 Yao-Chang Liu,8 Michael S. Torbenson,9 Aynur Unalp-Arida,3 Matthew Yeh,10 Arthur J. McCullough,11 and Arun J. Sanyal12 for the Nonalcoholic Steatohepatitis Clinical Research Network13. Design and Validation of a Histological Scoring System for Nonalcoholic Fatty Liver Disease. *Hepatology* **41**, 1313-1321 (2005).
